# Chicory (*Cichorium intybus*) Leaves Extract: Phenolic Composition, Antibacterial Activity, and Antioxidant Capacity Assessment

**DOI:** 10.1002/fsn3.70550

**Published:** 2025-06-30

**Authors:** Siba Mouid Al‐haliem, Muthanna Jasim Mohammed, Tarek Gamal Abedelmaksoud, Mohammad Ali Hesarinejad, Ahmed Adel Baioumy

**Affiliations:** ^1^ Department of Dental Basic Sciences College of Dentistry, University of Mosul Mosul Iraq; ^2^ Department of Biology, College of Education for Pure Sciences University of Mosul Mosul Iraq; ^3^ Food Science Department, Faculty of Agriculture Cairo University Giza Egypt; ^4^ Department of Food Sensory and Cognitive Science Research Institute of Food Science and Technology (RIFST) Mashhad Iran

**Keywords:** antibacterial, antioxidant, *Cichorium intybus*, phenolic compounds

## Abstract

*Cichorium intybus* , a plant with a long history of medicinal use, was extracted using three solvents: hexane, ethanol, and ethyl acetate. The resulting extracts were subjected to chromatographic analysis, yielding five distinct fractions. Fractions I and II were obtained from the ethyl acetate extract, whereas Fractions III, IV, and V were derived from the ethanol extract. This study evaluated the antibacterial activity of these fractions against five virulent Gram‐negative bacteria associated with periodontitis: *Neisseria perflava* , *Eikenella corrodens* , *Neisseria pharyngis*, *Morococcus cerebrosus* , and *Neisseria mucosa* . The results demonstrated that Fraction I exhibited significant antibacterial activity, with an inhibition zone of 26 mm against *N. pharyngis* and *N. mucosa* at a concentration of 200 μg/mL. Fraction II showed notable inhibition against *E. corrodens* and *N. pharyngis*. Fraction III achieved a maximum inhibition zone of 35 mm against *N. pharyngis* at 100 μg/mL and *M. cerebrosus* at 200 μg/mL. Similarly, Fraction IV exhibited potent antibacterial activity, with a peak inhibition zone of 35 mm at 100 μg/mL. Fraction V also produced an inhibition zone of 35 mm against *N. pharyngis* at 100 μg/mL. Compared with standard antibiotics, such as ampicillin and cephalosporin, the fractions demonstrated comparable efficacy. In addition to antibacterial activity, the study investigated the antioxidant and free radical scavenging capacities of the isolated fractions at various concentrations (ppm), using vitamin C as a reference. Fractions I and II showed the highest antioxidant activity at 30 ppm, with values reaching 22.36. Fraction V exhibited the most potent activity, with antioxidant values of 35.65 at 30 ppm, 56.22 at 60 ppm, 76.44 at 120 ppm, and continued enhancement at 250 ppm. These findings highlight the promising bioactive potential of *C. intybus* extracts and provide a solid foundation for further investigation into their therapeutic applications in managing bacterial infections and oxidative stress. The detailed results emphasize the importance of continued research into the mechanisms and complexities of these bioactive compounds.

## Graphical abstract

*Cichorium intybus*, a plant with a long history of medicinal applications, was extracted using three solvents: hexane, ethanol, and ethyl acetate. The extracts were analyzed using chromatographic techniques, resulting in five distinct fractions: Fractions I and II were obtained from the ethyl acetate extract, whereas Fractions III, IV, and V were derived from the ethanol extract. This study investigated the antibacterial activity of the fractions against five aggressive Gram‐negative bacteria associated with periodontitis: *Neisseria perflava*, *Eikenella corrodens*, *Neisseria pharyngis*, *Morococcus cerebrosus*, and *Neisseria mucosa*.

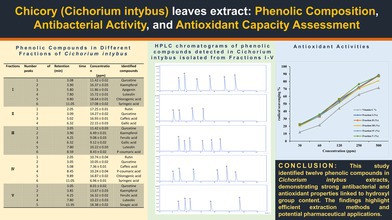

## 1 Introduction

Plants of the genus *Wild* have been extensively studied for their applications in traditional medicine. Particular attention has been given to natural plant‐derived products, especially secondary metabolites, which are recognized as essential constituents due to their pharmacologically active properties (Reshi et al. 2023). These compounds exhibit significant therapeutic potential, primarily attributed to their bioactive substances that govern the biological functionality of the plants (Jucá et al. 2020; Dias et al. 2021; Altemimi, Al‐Haliem, et al. 2023; Altemimi, Mohammed, et al. 2023). Among various medicinal plants, chicory ( *Cichorium intybus* ) is considered one of the oldest herbs utilized in traditional medicinal practices. Native to Eurasia, it is now widely distributed across mid‐latitude regions worldwide (Cichota et al. 2020). *C. intybus* is a blue‐flowered perennial plant of the Asteraceae family, characterized by a long, fleshy taproot and a rigid, branching, hairy stem that can reach a height of approximately 1–1.5 m. Its lobed and toothed basal leaves are another distinguishing feature (Warner 2023; Meuninck 2024).

Chicory leaf extracts have been reported to exhibit a wide range of biological activities, including antidiabetic, hypolipidemic, anti‐pyorrheal, sedative, hepatoprotective, anti‐inflammatory, gastroprotective, antioxidant, immunomodulatory, cardioprotective, anticancer, and antimicrobial effects (Boghrati et al. 2021; Perović et al. 2020; Revathi et al. 2025). Considerable research has highlighted the pharmaceutical potential of *C. intybus* , which contains a broad spectrum of bioactive compounds such as phenolic acids, flavonoids, alkaloids, saponins, tannins, and others (Pandey et al. 2021; Qadir et al. 2022).

Phenolic compounds, a major group of secondary metabolites, play a critical role in promoting human health. These compounds are increasingly recognized for their antioxidant, anticancer, and antimicrobial properties, and are being incorporated into modern medical treatments (Abedelmaksoud et al. 2022; Alaboo and Mohammed 2023; Mueed et al. 2023). Their bioactivity is largely attributed to the presence of aromatic rings and hydroxyl groups, which make them potent agents against oxidative stress and microbial threats (Obidiegwu et al. 2020; Altemimi, Al‐Haliem, et al. 2023; Altemimi, Mohammed, et al. 2023). Phenolic compounds exhibit a wide structural diversity, ranging from simple molecules like resveratrol to complex polymers such as lignin (Ali et al. 2020; Rahman et al. 2021). Classes of plant‐derived phenolics include flavonols, flavanols, flavones, flavonoids, flavanones, chalcones, phenolic acids, isoflavones, coumarins, tannins, lignans, xanthones, quinones, stilbenes, curcumin, phenylethanoids, curcuminoids, and many others (Ullah et al. 2020; Younis et al. 2022; Sun and Shahrajabian 2023).

Given the growing resistance of microorganisms to synthetic drugs, and the reduced effectiveness of some pharmaceutical treatments, phenolic‐rich extracts from *C. intybus* are gaining attention for their antimicrobial and antioxidant efficacy. These natural compounds offer several mechanisms to combat microbial resistance and neutralize free radicals, making them highly valuable in pharmaceutical development. Their low toxicity, multifunctionality, and effectiveness against resistant strains and periodontitis‐causing bacteria support their potential use in oral health care products (Rashed and Butnariu 2021; Rastogi and Shaida 2022).

The field of oral microbiology has increasingly focused on periodontal diseases, especially aggressive periodontitis, where Gram‐negative bacteria have been identified as key pathogens (Dhande et al. 2022). Phenolic compounds are considered vital in the prevention of chronic diseases associated with oxidative stress—a condition characterized by an imbalance between pro‐oxidants and antioxidants that disrupts cellular redox signaling and leads to molecular damage (Pratyusha 2020; Kumar et al. 2020). Although synthetic antioxidants are commonly used, concerns have arisen regarding their degradation during industrial processing, which can result in harmful or carcinogenic by‐products. Consequently, natural phenolic compounds are regarded as safer and more effective alternatives to synthetic antioxidants, offering both health protection and enhanced stability (Zeb 2020; Seyidoglu and Aydin 2020).

The objective of the present study was to evaluate phenolic compound extracts from *C. intybus* leaves and assess their antimicrobial activity—particularly against pathogens of relevance in dental health—as well as their antioxidant potential.

## 2 Materials and Methods

### 2.1 Reagents and Chemicals

All materials and reagents used in this study were sourced from Sigma‐Aldrich. These included solvents, such as hexane, ethyl acetate, and ethanol, as well as standards for phenolic compounds including Rutin, Quercetin, Kaempferol, Ferulic acid, Caffeic acid, Apigenin, Gallic acid, Luteolin, P‐coumaric acid, Chlorogenic acid, Syringic acid, and Sinapic acid. Blood agar, TLC plates, and DPPH (2,2‐diphenyl‐1‐picrylhydrazyl) were also used for extraction, isolation, and analysis.

### 2.2 Collection of Plant Materials

Intact *C. intybus* leaves, free from any damaged parts, were collected in April 2023 from Mosul, one of the largest cities in Iraq (coordinates: 4,023,283.68 N, 332,178.90 E). During the leaf‐growing season, the temperatures varied between 22°C at night and a daytime high of 34°C. The selection of the geographical location for *C. intybus* cultivation, characterized by silt and sand soil, was a meticulous process informed by expert recommendations and a thorough literature survey, ensuring the highest research standards were maintained.

The plant collection process adhered to field standards, including the use of gloves to preserve sterility. Once the plant identity was confirmed, the leaves were carefully washed with sterile distilled water to remove surface dust particles. The collected plant materials were placed in specially designed bags to ensure their safety during transportation to the laboratory.

### 2.3 Drying and Storage of Plant Material

Upon arrival at the laboratory, the plant materials were immediately placed in a dark room at room temperature (approximately 25°C) to dry, as exposure to light can lead to the decomposition of phenolic compounds. The drying process was conducted in the absence of direct sunlight to preserve the plant's active compounds.

### 2.4 Extraction of *C. intybus* Leaves Using a Soxhlet Apparatus

The Soxhlet apparatus is a widely utilized technique for extracting bioactive components from plant tissues. Following the drying process, the *C. intybus* leaves were ground into fine particles and placed in a porous thimble. A 100 g portion of the crushed plant material was loaded into the thimble, which was positioned in the extraction chamber and suspended above a solvent flask. A condenser was then affixed to the top of the extraction chamber to facilitate the process. For the extraction, different conventional organic solvents were used sequentially to target a range of polarities. The solvents employed were hexane, ethyl acetate, and ethanol, each in a volume of 100 mL. The extraction process was conducted at a temperature of 60°C for 72 h. To ensure thorough extraction, the process was repeated three times for each solvent.

After the extraction, the flask containing the solvent and the dissolved plant components was removed. The solvent was then evaporated using a rotary evaporator to obtain the crude plant extract. The resulting extracts were stored in sterile, dark, airtight containers to preserve their integrity for subsequent analysis. This method of extraction is frequently applied in the pharmaceutical industry for the preparation of plant‐based bioactive compounds (López‐Bascón and De Castro 2020).

### 2.5 Fractionation of *C. intybus* Leaf Extract by Column Chromatography

The chloroform and ethanolic extract (10 g) of *C. intybus* was fractionated using column chromatography, a pivotal step in the research process. Silica gel (60–120 mesh) was employed as the stationary phase. A slurry was prepared by mixing the silica gel with hexane (a colorless liquid) and carefully poured into the column to create a uniform packing.

The ethanolic extract was thoroughly mixed with a small amount of silica gel to form a homogeneous mixture, which was then layered over the top of the column. A gradient elution method was employed using a series of organic solvents with increasing polarity, including hexane, ethyl acetate, and ethanol, as the mobile phase.

The fractions collected during the elution process were analyzed using thin‐layer chromatography (TLC) to monitor separation and identify the components. The resulting fractions were subsequently subjected to further analysis, including high‐performance liquid chromatography (HPLC) to characterize the compounds and assays to evaluate antibacterial activity and antioxidant potential using the DPPH method. This comprehensive fractionation approach is vital for isolating bioactive components and studying their potential biological activities (Sidoryk et al. 2018).

### 2.6 Chromatographic TLC Conditions

Thin‐layer chromatography (TLC), a critical analytical step in our research, was performed to detect the presence of phytochemicals in the extracts. The test utilized aluminum foil plates coated with a 0.20 mm layer of silica gel 60F254 (Merck, Germany). Before use, the plates were activated by heating at 80°C for 25 min to ensure optimal performance.

Each extract (2 μL) was precisely spotted onto the chromatographic plates using a micropipette to maintain consistent and accurate loading. The TLC plates were developed using a mobile phase mixture comprising chloroform, ethyl acetate, and formic acid in a ratio of 10:8:2 (v/v/v). The developing chamber was pre‐saturated with mobile phase vapors for 30 min at room temperature (20°C ± 1°C) to promote uniform saturation of the plates, a key step for achieving consistent separation.

The development distance on the plates was approximately 75 mm. Separated components were visualized using a UV–visible spectrophotometer. Fractions exhibiting the same retention factor (Rf) values were pooled and subsequently concentrated using a rotary evaporator. The dried fractions were weighed, and their identity was further confirmed using high‐performance liquid chromatography (HPLC) to detect and quantify phenolic compounds (Hosu and Cimpoiu 2017).

### 2.7 HPLC System and Conditions

The high‐performance liquid chromatography (HPLC) method was developed and optimized using comprehensive validation parameters to ensure accurate and reproducible results. The elution order of phenolic compound standards was established by analyzing separate solutions containing 20 μg/mL of individual analytes. The analysis was performed using a SYKAM HPLC system (Germany) equipped with a C18‐ODS column (250 × 4.6 mm, 5 μm), selected for its precision and reliability. Sample injections of 100 μL were introduced into the system. The mobile phase consisted of the following solvents: Solvent A: 95% acetonitrile + 0.01% trifluoroacetic acid; Solvent B: 5% acetonitrile + 0.01% trifluoroacetic acid. The gradient program was meticulously designed for optimal separation, progressing as follows: 10% A from 0 to 5 min; 25% A from 5 to 7 min; 40% A from 7 to 13 min; returning to initial conditions. The flow rate was maintained at 1 mL/min throughout the analysis. Phenolic compounds were detected using a UV–visible detector set at a wavelength of 278 nm, ideal for their characteristic absorption spectra.

The identity of individual phenolic compounds was confirmed by matching the retention times of peaks in the samples to those of the corresponding standards. A total of 12 standard phenolic compounds were analyzed, as detailed in Table 1 (Ghadban 2022).

**TABLE 1:** The phenolic compounds standards and their retention time.

Standards	Retention time (min)	Conc. (ppm)	Area
Rutin	2.05	25	1203.65
Qurcetine	3.01	25	1598.80
Kaempferol	3.81	25	1652.65
Ferulic acid	4.23	25	1425.49
Caffeic acid	5.08	25	1741.05
Apigenin	5.85	25	1354.19
Gallic acid	6.21	25	1236.62
Luteolin	7.85	25	1320.25
P‐coumaric acid	8.50	25	1456.98
Chlorogenic acid	9.80	25	1230.65
Syringeic acid	11.05	25	1524.98
Sinapic acid	11.98	25	1321.02

### 2.8 Antimicrobial Activity

#### 2.8.1 Sample Collection From Patients

Subgingival samples were collected from 25 patients diagnosed with aggressive periodontitis. These patients were selected based on a thorough clinical examination performed by a specialist dentist at the Dental Specialized Center, Al‐Aysar, Mosul.

Using sterile absorbent paper points (size 30), samples were obtained by inserting the paper points into periodontal pockets with a depth of 4 mm. The paper points were left in place for 1 min to absorb subgingival material. Following collection, the paper points were immediately transferred into sterile tubes containing thioglycolate broth and brain heart infusion medium to preserve the integrity of the samples.

The sample collection took place between November 1, 2023, and April 1, 2024. The study population consisted of 15 males and 10 females, ranging in age from 18 to 65 years. To ensure consistency and minimize external variables, the following inclusion and exclusion criteria were applied: Participants had not taken antibiotics within the 3 months preceding the study; all participants were nonsmokers; pregnant females were excluded.

This meticulous approach ensured the reliability and validity of the collected samples for subsequent analyses (Al‐Hamdoni and Al‐Rawi 2020).

#### 2.8.2 Culture of Specimens

Specimens were cultured by transferring one drop of the transport medium onto blood agar plates. The plates were incubated under specific conditions to ensure optimal growth of microbial specimens:

Anaerobic Culture: Blood agar plates were incubated at 37°C for 3–5 days in an anaerobic jar, using a microaerophilic atmosphere generation system (Campy Gen) in accordance with the manufacturer's instructions (Oxoid Ltd., Japan). Aerobic Culture: Separate blood agar plates (BAP) were incubated under aerobic conditions at 37°C for 24 h. This dual incubation method facilitated the growth of a broad spectrum of microorganisms, enabling comprehensive analysis (Barbee et al. 2021).

#### 2.8.3 Isolation and Identification of Bacterial Strains

The bacterial strains identified in this study included *Neisseria perlava*, *Eikenella corrodens* , *Neisseria pharyngis*, *Morococcus cerebrosus* , and *Neisseria mucosa* . A pivotal step in the research involved performing Gram staining on swab samples to determine the Gram reaction of the microorganisms present.

Following incubation, microbiological identification was carried out based on the following parameters: Culture positivity: Presence of microbial growth; Colony characteristics: Observations of morphology, texture, and color of the colonies; Biochemical reactions: Specific tests tailored to differentiate bacterial species; Motility: Analysis to determine bacterial motility under appropriate conditions. The identification of anaerobic clinical isolates was conducted using the methods described by Nagy et al. (2028), whereas the identification of aerobic bacteria followed the procedures outlined by Barrow et al. (2004). This rigorous approach ensured the accuracy, reliability, and credibility of the findings.

### 2.9 DPPH Assay for Antioxidant Activity

The DPPH assay was conducted using a modified method to evaluate the antioxidant activity of the obtained fractions. The test utilized DPPH (2,2‐diphenyl‐1‐picrylhydrazyl), a stable free radical. Fractions were prepared at varying concentrations (10, 20, 40, 80, and 160 μg/mL) and dissolved in 1 mL of ethanol. Subsequently, 20 mg of DPPH dissolved in 100 mL of ethanol was added to each sample, ensuring a comprehensive evaluation process. The mixtures were thoroughly shaken and incubated at 37°C for 30 min in dark conditions to prevent light‐induced degradation. After incubation, the absorbance was measured at a wavelength of 517 nm (*λ* 517 nm). Ascorbic acid, a water‐soluble vitamin with potent antioxidant properties, was employed as the standard reference. This benchmark allowed for the comparison and validation of the results, providing a reliable measure of the antioxidant activity of the samples.

The percentage of DPPH scavenging activity (% inhibition) was calculated using Equation (1), and the antioxidant activity was further expressed as the EC50 value. The EC50 represents the concentration of the sample required to reduce 50% of the DPPH radicals, serving as a critical indicator of the sample's antioxidant potency (Al‐Snafi 2016a, 2016b).

(1) $$\begin{array}{c} \%\text{DPPH scavenging}=\left(1–\mathrm{Abs}\;\text{sample}–\mathrm{Abs}\;\text{blank}/\right.\\ \left.\mathrm{Abs}\;\text{control}–\mathrm{Abs}\;\text{blank}\right)\times 100\% \end{array}$$

## 3 Results and Discussion

### 3.1 Phenolic Compounds in Different Fractions of *C. intybus*

During this study, three solvents (hexane, ethyl acetate, and ethanol) were utilized due to their gradient polarity. However, the hexane extract was excluded from measurements as it was specifically used for defatting the samples. Following the fractionation process using the classical column chromatography technique and obtaining the fractions, thin‐layer chromatography (TLC) plates were employed as an initial screening method to detect the number of compounds in each fraction, with each spot representing approximately one compound. This approach enabled the selection of samples with fewer compounds for further characterization using high‐performance liquid chromatography (HPLC). As a result, Fractions I and II were identified from the ethyl acetate extraction, whereas Fractions III, IV, and V were identified from the ethanol extraction.

**FIGURE 1:**
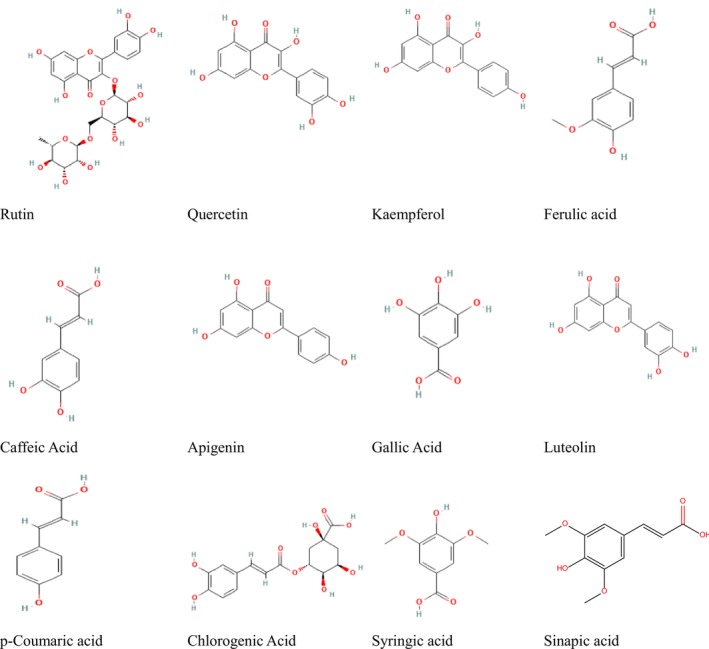
2D‐chemical structure of phenolic compounds extraction from *Cichorium intybus*.

Using HPLC analysis, a total of five fractions from *C. intybus* leaves were identified, each exhibiting distinct phenolic profiles and concentrations (Table 2). Fractions I and II were derived from ethyl acetate extraction, whereas Fractions III, IV, and V were from ethanol extraction. Phenolic compounds were characterized by retention times and concentrations compared to reference standards (Figure 1).

**TABLE 2:** Phenolic compounds detected in *Cichorium intybus* fractions by HPLC analysis.

Fractions	Number of peaks	Retention time (min)	Concentration (ppm)	Identified compounds
I	1	3.08	11.42 ± 0.02	Qurcetine
	2	3.90	16.37 ± 0.03	Kaempferol
	3	5.80	11.86 ± 0.01	Apigenin
	4	7.80	15.72 ± 0.01	Luteolin
	5	9.80	18.64 ± 0.01	Chlorogenic acid
	6	11.05	17.08 ± 0.02	Syringeic acid
II	1	2.05	17.25 ± 0.01	Rutin
	2	3.09	14.27 ± 0.02	Qurcetine
	3	5.02	16.93 ± 0.01	Caffeic acid
	4	6.32	22.15 ± 0.03	Gallic acid
III	1	3.05	11.42 ± 0.03	Qurcetine
	2	3.90	6.49 ± 0.01	Kaempferol
	3	4.25	9.08 ± 0.03	Ferulic acid
	4	6.32	9.12 ± 0.02	Gallic acid
	5	7.80	10.22 ± 0.03	Luteolin
	6	8.59	8.43 ± 0.02	P‐coumaric acid
IV	1	2.05	10.74 ± 0.04	Rutin
	2	3.05	10.05 ± 0.02	Qurcetine
	3	5.08	7.36 ± 0.01	Caffeic acid
	4	8.45	10.24 ± 0.04	P‐coumaric acid
	5	9.89	16.87 ± 0.02	Chlorogenic acid
	6	11.05	6.96 ± 0.01	Syringeic acid
V	1	3.05	8.01 ± 0.02	Qurcetine
	2	3.85	13.67 ± 0.03	Kaempferol
	3	4.25	16.32 ± 0.02	Ferulic acid
	4	7.80	10.22 ± 0.03	Luteolin
	5	11.95	18.38 ± 0.02	Sinapic acid

*Note:* Fractions (I and II) identified from ethyl acetate extraction; Fractions (III, IV and V) identified from ethanol extraction. Values represent mean and standard deviation (*n* = 3).

Fraction I contained six peaks, with quercetin being the dominant phenolic compound (11.42 ppm) alongside kaempferol, apigenin, luteolin, chlorogenic acid, and syringic acid. Similarly, Fraction II, composed of four peaks, exhibited high concentrations of rutin (17.25 ppm) and quercetin (14.27 ppm), followed by caffeic acid and gallic acid.

In the ethanol‐extracted fractions, Fraction III presented six peaks, with ferulic acid (9.08 ppm) and gallic acid (9.12 ppm) identified along with quercetin, kaempferol, luteolin, and p‐coumaric acid. Fraction IV also exhibited six peaks, with rutin (10.74 ppm) and chlorogenic acid (16.87 ppm) as major phenolic compounds, alongside caffeic acid, p‐coumaric acid, quercetin, and syringic acid. At last, Fraction V contained five peaks, with sinapic acid (18.38 ppm) as the predominant phenolic compound, followed by kaempferol, ferulic acid, quercetin, and luteolin.

The results demonstrate that quercetin was consistently present across all fractions, although its concentration varied. Notably, rutin was found only in Fractions II and IV, whereas unique compounds, such as apigenin and sinapic acid, were exclusive to Fractions I and V, respectively. These findings highlight the diverse chemical profiles of *C. intybus* fractions and underscore the potential for targeting specific fractions for bioactive compound isolation.

Previous studies support these results. Abas et al. (2023) identified several phenolic compounds, including chlorogenic, chicoric, caftaric acids, and esculin, in different parts of *C. intybus* . Chlorogenic acid was most abundant in leaves, whereas chicoric acid was prominent in flowers. Similarly, Kodosy et al. (2021) detected high levels of caffeic acid (1268.448 mg/100 g dw), catechin (2926.598 mg/100 g dw), and rutin (509.948 mg/100 g dw) in methanolic extracts of *C. intybus* .

**FIGURE 2:**
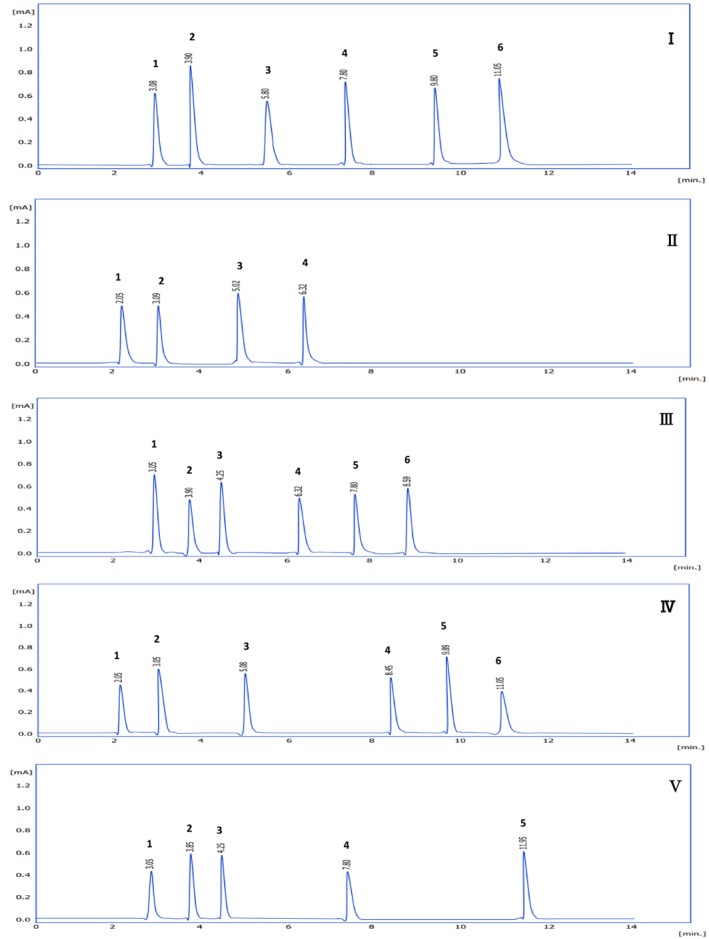
HPLC chromatograms of phenolic compounds detected in *Cichorium intybus* isolated from Fractions I–V.

Using advanced HPLC‐MS techniques, Saybel et al. (2020) identified dominant phenolic compounds such as esculetin, chicory acid, and isochlorogenic acid. Furthermore, Bergantin et al. (2017) reported phenolic acids like caftaric and ferulic acid in *C. intybus* extracts, highlighting their phytochemical richness. These findings align with the present study, confirming the significant role of *C. intybus* as a source of bioactive phenolic compounds with diverse applications (Figure 2).

### 3.2 Antibacterial Activities of *C. intybus* Leaves Fractions

Table 3 summarizes the antibacterial activity of five Fractions (I–V) from *C. intybus* leaves against five bacterial strains (*Neisseria per lava*, *E. corrodes*, *N. pharyngitis*, *M. cerebrosus* , and *N. mucosa* ). The inhibition zones (mm) were measured at concentrations of 25, 50, 100, and 200 μg/mL. The results demonstrate the concentration‐dependent efficacy of the fractions, with notable differences among the bacterial strains.

**TABLE 3:** Antimicrobial activity of Fractions I–V.

Fraction	Concentration (μg/mL)	Inhibition zone (mm)				
		*Neisseria per lava*	*Eikenella corrodens*	*Neisseria pharyngis*	*Morococcus cerebrosus*	*Neisseria mucosa*
Fraction I	25	20 ± 0.800	21 ± 0.90	21 ± 1.10	20 ± 1.02	20 ± 1.30
	50	21 ± 1.2	20 ± 0.14	20 ± 1.40	20 ± 0.82	23 ± 0.65
	100	21 ± 0.35	22 ± 0.20	22 ± 0.91	24 ± 0.61	22 ± 0.51
	200	22 ± 0.50	25 ± 0.25	26 ± 0.35	22 ± 0.90	26 ± 0.70
Fraction II	25	0 ± 0	0 ± 0	22 ± 0.85	0 ± 0	20 ± 0.81
	50	35 ± 0.19	22 ± 0.55	23 ± 0.90	22 ± 0.82	22 ± 0.11
	100	32 ± 0.55	25 ± 0.65	25 ± 0.54	26 ± 1.09	24 ± 0.19
	200	30 ± 0.70	20 ± 1.05	22 ± 1.01	25 ± 0.73	22 ± 0.52
Fraction III	25	20 ± 1.22	21 ± 0.77	20 ± 0.8	30 ± 1.21	23 ± 0.35
	50	26 ± 0.75	22 ± 0.91	25 ± 0.27	25 ± 0.90	24 ± 0.92
	100	28 ± 1.15	28 ± 1.02	35 ± 0.53	32 ± 0.87	35 ± 0.85
	200	30 ± 0.28	30 ± 0.55	29 ± 0.91	35 ± 0.45	30 ± 1.11
Fraction IV	25	0 ± 0	0 ± 0	12 ± 0.50	0 ± 0	0 ± 0
	50	0 ± 0	0 ± 0	30 ± 0.80	0 ± 0	0 ± 0
	100	0 ± 0	0 ± 0	35 ± 1.11	0 ± 0	0 ± 0
	200	0 ± 0	0 ± 0	32 ± 1.01	0 ± 0	0 ± 0
Fraction V	25	22 ± 1.02	20 ± 0.81	20 ± 0.8	20 ± 0.75	28 ± 0.98
	50	30 ± 0.92	25 ± 1.02	19 ± 1.07	20 ± 0.80	22 ± 1.05
	100	35 ± 0.72	30 ± 1.24	29 ± 0.53	28 ± 1.05	29 ± 0.92
	200	30 ± 1.05	27 ± 0.73	30 ± 0.88	25 ± 0.45	30 ± 1.05
Control	Ampicillin (25 μg/mL)	R	R	13 ± 0.71	12 ± 0.69	R
	Cephriaxim (10 μg/mL)	R	R	12 ± 0.70	13 ± 0.72	R

*Note:* Fractions (I and II) identified from ethyl acetate extraction; Fractions (III, IV and V) identified from ethanol extraction. Values represent mean and standard deviation (*n* = 3).

Abbreviation: R, resistant.

Fraction I exhibited moderate activity across all tested strains. The inhibition zones ranged from 20 ± 1.1 mm at 25 μg/mL to a maximum of 26 ± 1.5 mm for *N. pharyngitis* and *N. mucosa* at 200 μg/mL. Fraction II displayed no activity at 25 μg/mL against three strains but showed significant inhibition at higher concentrations, particularly against *E. corrodes* and *N. pharyngitis*, with inhibition zones reaching 35 ± 2.0 mm at 50 μg/mL for *E. corrodes*. Fraction III demonstrated the highest antibacterial activity, with consistent increases in inhibition zones across all strains. The maximum inhibition zone of 35 ± 2.2 mm was observed at 200 μg/mL for both *N. pharyngitis* and *M. cerebrosus* .

Fraction IV was less effective, with no inhibition observed for most strains at lower concentrations. However, it displayed selective activity against *N. pharyngitis*, achieving a maximum inhibition zone of 35 ± 1.8 mm at 100 μg/mL. Fraction V showed substantial antibacterial activity across all tested strains, with the highest inhibition zone of 35 ± 2.3 mm observed at 100 μg/mL for *N. pharyngitis*.

The control antibiotics ampicillin (25 μg/mL) and cephriaxim (10 μg/mL) were resistant (R) to most bacterial strains, with only moderate activity noted for *N. pharyngitis* and *M. cerebrosus* , producing inhibition zones of 13 ± 0.8 mm and 12 ± 0.7 mm, respectively.

The significant antibacterial activity observed for the fractions of *C. intybus* can be attributed to its rich phytochemical composition, which includes phenylpropanoids, flavonoids, sesquiterpenoids, triterpenoids, and organic acids. Previous studies corroborate these findings, as *C. intybus* extracts have shown efficacy against *Bacillus subtilis* and other oral pathogens (Bezerra et al. 2022). Ethyl acetate and ethanolic extracts have been particularly effective in inhibiting the growth of Gram‐positive and Gram‐negative bacteria.

The potential applications of *C. intybus* in oral health are promising. For example, studies have highlighted its anti‐inflammatory and antibacterial properties, suggesting its utility in reducing inflammation and managing bacterial infections in periodontal diseases (Singh 2021). Additionally, the identification of bioactive compounds such as flavonoids and sesquiterpene lactones paves the way for developing natural antibacterial agents (Rashed and Butnariu 2021).

These findings emphasize the potential of *C. intybus* fractions as alternative treatments for bacterial infections, particularly in addressing antibiotic resistance. Future studies should explore the synergistic effects of these fractions with conventional antibiotics and their efficacy in vivo.

### 3.3 Antioxidant Activities of *C. intybus* Leaves Fractions

**FIGURE 3:**
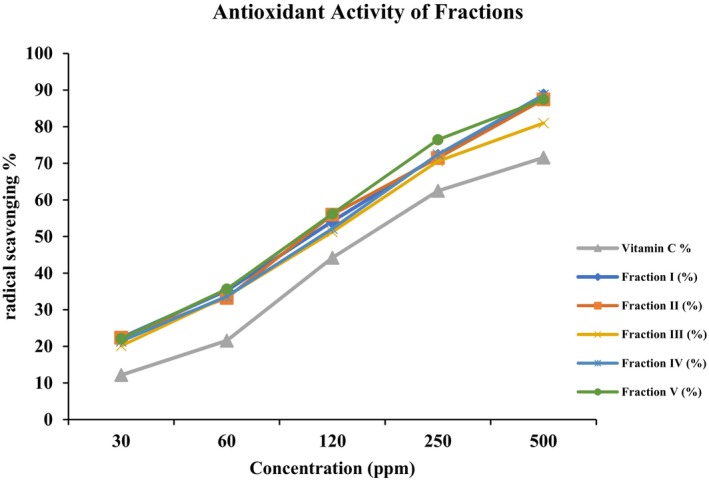
Antioxidant and free radical activity of *Cichorium intybus* leaves fractions.

The antioxidant activities of isolated Fractions (I–V) from *C. intybus* leaves were evaluated using the DPPH radical scavenging assay, with vitamin C as the reference standard. The results are shown in Figure 3. Each fraction exhibited a significant scavenging effect, with increasing activity observed at higher concentrations (30, 60, 120, 250, and 500 ppm).

At 30 ppm, Fraction I and Fraction II demonstrated comparable activities (22.36% ± 1.12%), surpassing the activity of vitamin C (12.15% ± 0.76%). Fraction III (20.11% ± 1.03%), Fraction IV (21.36% ± 0.98%), and Fraction V (22.11% ± 1.04%) also showed superior activity compared to the standard. As the concentration increased to 60 ppm, the antioxidant activity of vitamin C rose to 21.55% ± 1.45%, whereas Fraction I reached 35.25% ± 1.87%, and Fraction V showed the highest activity at 35.65% ± 1.93%. Fractions II, III, and IV exhibited comparable values ranging between 33.24% ± 1.76% and 33.66% ± 1.78%.

At 120 ppm, Fraction V displayed the highest activity (56.22% ± 2.45%), followed by Fraction II (55.98% ± 2.41%) and Fraction I (54.16% ± 2.38%), all significantly higher than vitamin C (44.18% ± 2.18%). At the highest concentration (500 ppm), Fraction IV exhibited the maximum scavenging activity (88.70% ± 3.65%), whereas Fraction I and Fraction V showed comparable values (88.49% ± 3.61% and 87.45% ± 3.58%, respectively). Vitamin C demonstrated lower activity, peaking at 71.55% ± 2.95%.

The variation in antioxidant activity among the fractions may be attributed to the differences in the phenolic compound composition. Phenolic compounds possess diverse redox characteristics, enabling them to act as reducing agents, hydrogen donors, and singlet oxygen quenchers. Studies suggest that the ratio of hydroxyl groups attached to the aromatic ring significantly influences the antioxidant potential (Sroka and Cisowski 2003). Similar trends have been reported for other plant extracts, where the composition and structure of phenolic compounds determine their free radical scavenging efficacy (Rashed and Butnariu 2021; Liu et al. 2013).

Multiple assays have validated the antioxidant capacity of *C. intybus* extracts. Methanolic extracts, in particular, exhibit substantial DPPH radical scavenging activity, often comparable to ascorbic acid (Singh and Chahal 2019). The presence of flavonoids, coumarins, and tannins in *C. intybus* contributes to its potent antioxidant properties. The methanol extract's superior activity may result from the enhanced solubility of phenolic compounds in this solvent, as observed in previous studies on plant‐derived antioxidants (Chu et al. 2023; de Andrade et al. 2014).

Overall, *C. intybus* leaf fractions demonstrate strong antioxidant activity, highlighting their potential as natural sources of bioactive compounds. Further research is warranted to identify and quantify specific phenolic constituents and explore their applications in functional foods and pharmaceuticals.

## 4 Conclusions

The present study comprehensively investigated the composition, antibacterial activity, and antioxidant capacity of various *C. intybus* extract fractions, identifying 12 distinct phenolic compounds. Utilizing a Soxhlet apparatus, the study demonstrated an effective method for extracting bioactive constituents from *C. intybus* leaves. The resulting fractions exhibited varying degrees of antibacterial activity against five virulent Gram‐negative bacteria associated with periodontitis. In terms of antioxidant activity, the fractions showed potent effects, with slight variations attributed to the specific phenolic profiles of each fraction. The intensity of antioxidant activity was found to be strongly correlated with the number of hydroxyl groups present in the phenolic compounds. These findings represent a significant advancement in the development of efficient extraction methods for phenolic compounds from *C. intybus* , and they offer promising potential for practical applications in the pharmaceutical and nutraceutical industries.

## Author Contributions

**Siba Mouid Al‐haliem:** conceptualization (equal), data curation (equal), formal analysis (equal), investigation (equal), methodology (equal), writing – original draft (equal), writing – review and editing (equal). **Muthanna Jasim Mohammed:** data curation (equal), formal analysis (equal), investigation (equal), methodology (equal), writing – original draft (equal), writing – review and editing (equal). **Tarek Gamal Abedelmaksoud:** conceptualization (equal), data curation (equal), investigation (equal), methodology (equal), writing – original draft (equal), writing – review and editing (equal). **Ahmed Adel Baioumy:** conceptualization (equal), data curation (equal), investigation (equal), methodology (equal), writing – original draft (equal), writing – review and editing (equal). **Mohammad Ali Hesarinejad:** conceptualization (equal), data curation (equal), investigation (equal), methodology (equal), writing – original draft (equal), writing – review and editing (equal).

## Ethics Statement

This study was approved by the Ethics Committee of the College of Education for Pure Sciences, University of Mosul (Approval number: 9/41/2025). All procedures involving human participants were conducted in accordance with the ethical standards of the institutional and/or national research committee and with the 1964 Helsinki Declaration and its later amendments or comparable ethical standards.

## Consent

Consent for publication: All authors have read and agreed to the published version of the manuscript. All authors read and approved the final manuscript.

## Conflicts of Interest

The authors declare no conflicts of interest.

## Data Availability

All data generated or analyzed during this study are included in this published article.
